# Pulmonary Hilar Cavernoma

**DOI:** 10.5334/jbsr.3860

**Published:** 2025-04-30

**Authors:** Roxana Ternoveanu, Laurent Médart, Malek Tebache

**Affiliations:** 1 Intern in Radiology, Radiology Department, Hôpital de la Citadelle de Liège, Belgium; 2 Radiologist, Hôpital de la Citadelle de Liège, Belgium

**Keywords:** Pulmonary vein thrombosis, Pulmonary hilar cavernoma

## Abstract

*Teaching point:* Description of a new radiological sign named “pulmonary hilar cavernoma” in a case of unilateral total pulmonary vein thrombosis.

## Case History—Clinical and Radiological Features

A 64‑year‑old man without fever was referred for angio‑chest computed tomography (angio‑chest CT) for productive cough, hemoptysis, and left‑sided chest pain for weeks. The patient had a history of recurring paroxysmal atrial fibrillation and successfully underwent pulmonary vein isolation by radiofrequency ablation 3 months earlier in another hospital, without notable complication.

Two months prior to the current assessment, an unenhanced chest CT examination (not shown) was performed at another hospital, suggestive of left pneumonia with pleural effusion without any improvement after antibiotic treatment. Bronchoscopy revealed an inflammatory mucosa, prone to bleeding. A single bronchial biopsy was performed and led to hemorrhage without clinical deterioration, suggesting well‑vascularized tissue. Analysis of the samples and blood ruled out infection.

**Figure 1:**
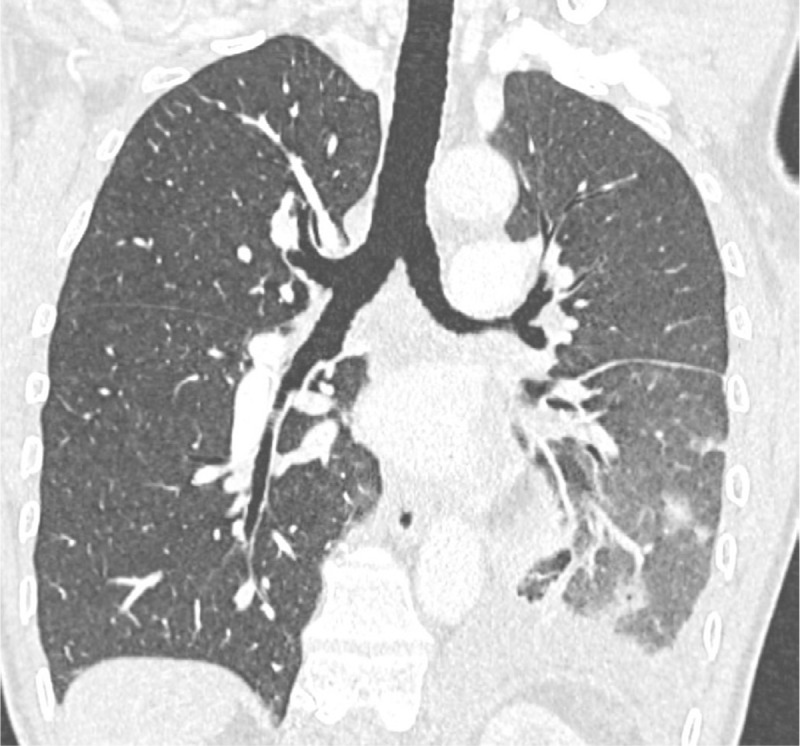
Contrast‑enhanced coronal chest CT (pulmonary window) with unilateral left signs of patchy alveolar hemorrhages, pulmonary edema, and pleural effusion.

**Figure 2:**
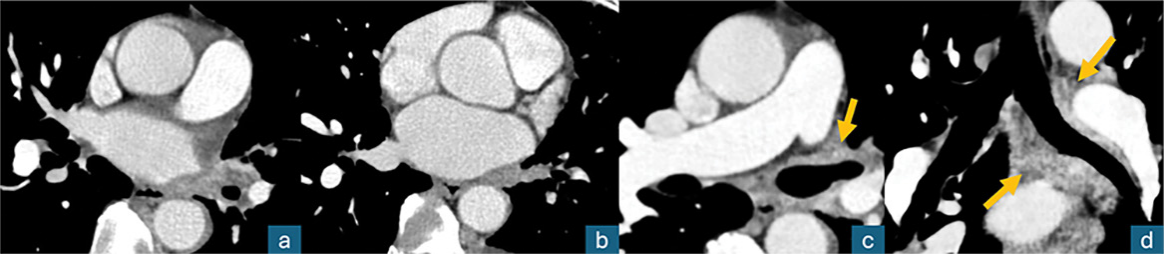
Contrast‑enhanced axial chest CT (mediastinal window); a, b: thrombosis of the left superior and inferior pulmonary veins; c, d: diffuse left peribronchial contrast‑enhancing infiltration (arrows), “pulmonary hilar cavernoma.”

**Figure 3:**
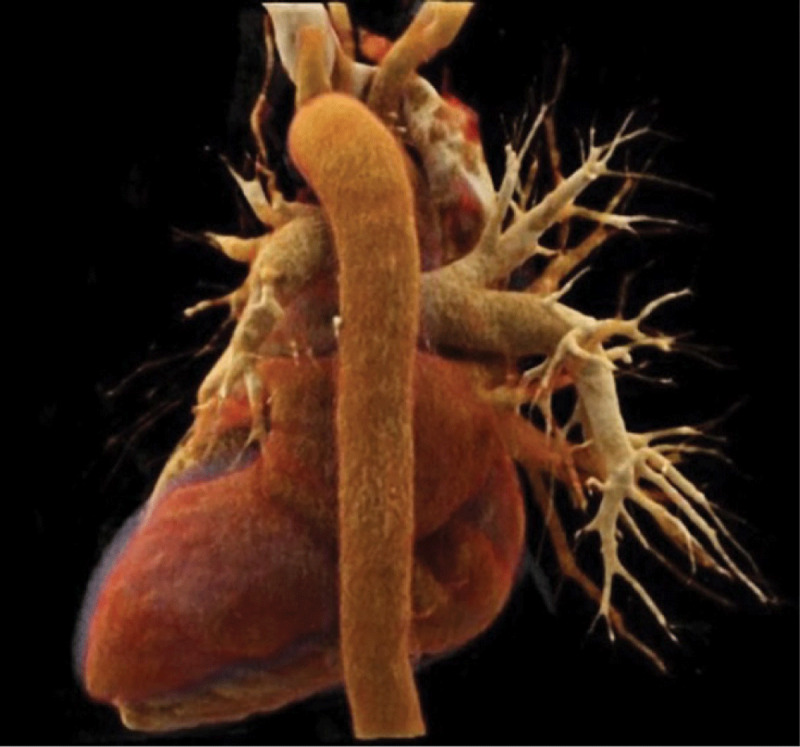
Cinematic VRT posterior view of the mediastinum; neither upper nor lower left pulmonary veins (in red color) are patent.

The present nongated angio‑CT illustrated unilateral areas of ground‑glass opacities associated with interlobular septa thickening and pleural effusion on the left side (Figure 1). These findings were observed in conjunction with thrombosis of both the superior and the inferior left pulmonary veins (Figures 2a, b and 3) and were therefore considered consistent with patchy alveolar hemorrhages and unilateral pulmonary edema in this context. A diffuse contrast‑enhancing infiltration of the left peribronchial tissue was also demonstrated, evocative of a collateral network of tiny veins recruited in the left hilar fat (Figure 2c, d). Angioplasty and stenting of the left inferior pulmonary vein was performed in a specialized center with a successful clinical outcome. The left superior pulmonary vein could not be recanalized.

## Commentary

Pulmonary vein thrombosis is a rare condition that may be associated with iatrogenic causes, such as lung transplantation or lobectomy, or may develop following pulmonary vein isolation procedures. Presentation is mostly asymptomatic; symptoms, if present, remain aspecific (cough, hemoptysis, and dyspnea), and the diagnosis is in many cases obtained by contrast‑enhanced chest CT [1].

Complications of pulmonary vein isolation by radiofrequency ablation include failure, groin hematoma, cerebral ischemic events, cardiac tamponade, and, in up to 5% of cases, pulmonary vein stenosis. The reduction in size and flow may lead to thrombosis of the stenosed pulmonary veins [1].

In this unfortunate case, the entire left lung venous drainage was compromised by thrombosis of the two pulmonary veins, stimulating the development of an extensive compensatory collateral central venous network bypassing the obstruction. These anomalous peribronchial collateral veins, however, remain functionally rather inadequate, leading to unilateral pulmonary edema and alveolar hemorrhage due to venous congestion and increased vascular pressure.

The formation of this collateral venous network following thrombosis of the left lung venous outflow system resembles the pathophysiological mechanism underlying the development of a portal cavernoma in the setting of portal vein thrombosis. Therefore, we propose to refer to this rare aspect as “pulmonary hilar cavernoma.”

## References

[r1] Chaaya G, Vishnubhotla P. Pulmonary vein thrombosis: a recent systematic review. Cureus. 2017;9(1):e993. 10.7759/cureus.993. 28265529 PMC5323025

